# Relationship of Homocysteine With Gender, Blood Pressure, Body Mass Index, Hemoglobin A1c, and the Duration of Diabetes Mellitus Type 2

**DOI:** 10.7759/cureus.19211

**Published:** 2021-11-02

**Authors:** Aqil Noor, Mahboob Ur Rahman, Noor Faraz, Kashif A Samin, Hamid Ullah, Amjad Ali

**Affiliations:** 1 Endocrinology, Hayatabad Medical Complex, Peshawar, PAK; 2 Medicine, District Head Quarter Hospital, Peshawar, PAK; 3 Cardiology, Lady Reading Hospital Medical Teaching Institution, Peshawar, PAK; 4 Family Medicine, Khyber Medical University, Peshawar, PAK; 5 Medicine, Bacha Khan Medical College, Mardan, PAK

**Keywords:** hyperhomocysteinemia, hba1c, blood pressure, diabetes, hemoglobin, body mass index, hcy

## Abstract

Introduction

Increased levels of homocysteine (Hcy) may lead to endothelial damage and increase the risk of cardiovascular and renal malfunction. The current study aimed to evaluate the association of serum Hcy levels with gender, body mass index (BMI), duration of diabetes mellitus type 2 (DMT2), hemoglobin A1c (HbA1c), and blood pressure (BP).

Methodology

A prospective observational study was conducted at Hayatabad Medical Complex in Peshawar, Pakistan in the department of endocrinology from June 2020 to June 2021. All patients with diagnosed DMT2 above the age of 18 years were included in the study. Individuals with unconfirmed diagnoses with ages over 75 years were excluded from the study. All data including the patient's age, gender, and medical history were recorded. Height and weight were used to calculate the BMI. BP was examined thrice and a mean value was recorded for each patient. For laboratory investigation, a vial of 3 ml blood was extracted keeping sterile and aseptic conditions by a trained nurse. The sample was sent for the determination of HbA1c and serum Hcy levels. Measurement of serum Hcy was done by chemiluminescent microparticle immunoassay. All data were documented by the researchers on a predefined pro forma.

Results

A total of 188 patients with DMT2 were included in the study with a mean age ± SD of 54.65 ± 8.42 years. Normal (<15 micromoles per liter [mcmol/l]) serum Hcy levels were reported in 75 (39.89%) individuals, while in 47 (41.59%) individuals, there was severe (>100 mcmol/l) hyperhomocysteinemia. More than half of the patients, i.e. 157 (83.52%), had HbA1c of greater than 7%, which indicated poor glycemic control. The study revealed that the majority of the female patients, i.e. 37 (78.72%), had severe hyperhomocysteinemia (p<0.0001). Similarly, there was a direct correlation between HbA1c levels and serum Hcy. Severe hyperhomocysteinemia was found in over 80% of the patients with poor glycemic control, i.e. HbA1c >7% (p<0.0001). Furthermore, the duration of DMT2 and hypertension were both significantly associated with increased levels of Hcy with p-values of <0.0001 and <0.0001, respectively. However, no association was found between hyperhomocysteinemia and BMI.

Conclusion

The study revealed that increased levels of serum Hcy were associated with female gender, poor glycemic control (HbA1c >7%), BP, and duration of DMT2. However, the study failed to find an association between serum Hcy and BMI. It is recommended that patients with poor glycemic control or those with the duration of DMT2 of more than five years must be regularly checked for hyperhomocysteinemia and renal function tests.

Large-scale and multi-center studies are required in order to determine the validity of these findings. The current study suggests that patients with diabetes mellitus and hypertension are likely to have increased levels of Hcy and, therefore, must be regularly screened for hyperhomocysteinemia.

## Introduction

According to the World Health Organization (WHO), the percentage of individuals with diabetes mellitus type 2 (DMT2) was 2.8% in 2000 and would increase to 4.4% by the year 2030. The increase in DMT2 cases from 171 million to 366 million in 30 years of duration since the last few decades was projected [1,2]. In 1995, WHO survey found that Pakistan ranked eighth among the 10 countries with the highest prevalence of diabetes. The same survey estimated that Pakistan will rank fourth with 14.5 million people with diabetes in 2025 [3]. In 2012, seven million people had diabetes [4]. The prevalence of DMT2 in Pakistan in 2015 reached 7.89% [5]. In other Southeast Asian countries, the percentage of patients with DMT2 varied greatly in the year 2014: Mauritius had a prevalence of 14.8%, India with 9.1%, Sri Lanka with 7.6%, Bangladesh with 6.3%, Bhutan with 5.8%, Nepal with 4.9%, and Maldives with 4.8% [6]. The risk of developing peripheral neuropathy was related to age, hemoglobin A1c (HbA1c), duration of DMT2 as well as retinopathy or neuropathy [7].

Hyperhomocysteinemia is common in patients with renal failure because proper kidney function is critical to homocysteine (Hcy) ​​metabolism [8,9]. Hyperhomocysteinemia increases the manufacturing of homocysteine thiolactone, oxidation products, Hcy ​​thiolactone, and mixed Hcy ​​disulfides thereby damaging endothelium by excessive connective tissue sulfation [10,11]. Ambrosch et al. showed an association of neuropathy with serum Hcy [12]. This connection was, however, not proved by other studies [13,14]. In addition, more literature have shown a connection between higher levels of plasma and the prevalence of other complications such as retinopathy and nephropathy, particularly in patients with DMT2 [13,15]. Other studies show that Hcy leads to endothelial damage. Normal levels of Hcy are less than 15 micromoles per liter (mcmol/l); however, higher levels are responsible for endothelial damage, which can subsequently lead to coagulopathy, coronary artery disease, myocardial infarction, and strokes. Hcy metabolism plays a major role in the pathogenesis of microvascular complications in patients with DMT2 [16].

The literature of this part of the developing countries of the world seems deficient. Comprehensive and explorative research on this topic has not been done recently. Therefore, our purpose of the study was to find out the association of serum Hcy levels with gender, HbA1c, blood pressure (BP), body mass index (BMI), and duration of DMT2.

## Materials and methods

A prospective observational study was conducted at Hayatabad Medical Complex (HMC) in Peshawar, Pakistan in the department of endocrinology from June 2020 to June 2021. Prior to the study being conducted, ethical approval was obtained from the institutional review board of HMC # IRB/26164/HMC. All diagnosed cases of DMT2, irrespective of gender and age above 18 years, were included in the study. All individuals with diabetes mellitus type 1, unconfirmed diagnosis of DMT2, age above 75 years, and those taking vitamin B supplements were excluded from the study. Informed verbal and written consent was obtained after the objectives and significance of the study were narrated to all the participants. 

All data including the patient's age, gender, and medical history were recorded. Height and weight were used to calculate the BMI using the following formula: weight (kg)/[height (meters)]^2^. The patients were divided according to the categories of BMI, i.e. underweight (<18 kg/m^2^), normal weight (18-24.9 kg/m^2^), overweight (>25.0-29.9 kg/m^2^), and obesity (30.0 kg/m^2^ and above). BP was examined thrice using the auscultatory method and with the aid of a sphygmomanometer and a stethoscope. The mean of three values was recorded for each patient. The patients were labeled as hypertensive if the systolic BP was over 140 mmHg and the diastolic was over 90 mmHg on at least two different occasions. 

For laboratory investigation, a vial of 3 ml blood was extracted keeping sterile and aseptic conditions by a trained nurse. Patients were instructed to fast before arrival for the blood sample extraction. The sample was sent for the determination of HbA1c and Hcy levels. HbA1c was determined by centrifugation in a Beckman Allegra Tm 6R centrifuge at 3000 revolutions per minute for at least 10-15 minutes. Once the plasma was separated, it was shifted into Eppendorf tubes and was frozen at -80°C for analysis of the Hcy. Measurement of Hcy was done by chemiluminescent microparticle immunoassay. The HbA1c of greater than 7% was labeled as poor glycemic control and the HbA1c of less than 7% indicated good glycemic control. The serum Hcy levels were categorized into the following: normal (<15 mcmol/l), moderate (15-30 mcmol/l), intermediate (30-100 mcmol/l), and severe (>100 mcmol/l). 

The data regarding sociodemographics, clinical history, and laboratory investigations were documented in a predefined pro forma. Data analysis was performed using Statistical Package for Social Sciences (SPSS version 23 [IBM Corp, Armonk, NY]). For continuous variables (BMI, age, Hcy levels, BP, HbA1c, and duration of DMT2), the mean ± SD were determined. For categorical variables (gender, categories of BMI, HbA1c, etc.), frequencies and percentages were calculated. The chi-squared test was applied to find out the association of Hcy levels with BP, BMI, and duration of DMT2. A p-value of less than or equal to 0.05 was taken as the cutoff for statistical significance. 

## Results

A total of 188 patients with DMT2 were included in the study with a mean age ± SD of 54.65 ± 8.42 years. The baseline characteristics of the study participants are presented in Table 1. 

**Table 1:** Characteristics of Study Patients (n=188) DMT2: diabetes mellitus type 2

Characteristics	Mean ± SD
Age (in years)	54.65 ± 8.42
Body mass index (in kg/m^2^)	29.5 ± 5.02
Systolic blood pressure (mmHg)	139 ± 21.78
Diastolic blood pressure (mmHg)	88.73 ± 14.70
Serum homocysteine (in mcmol/l)	92.45 ± 15.52
Duration of DMT2 (in years)	7.69 ± 6.76

There was female dominance with a frequency of 108 (57.44%). Normal (<15 mcmol/l) serum Hcy levels were reported in 75 (39.89%) individuals, while in 47 (41.59%) individuals, there was severe (>100 mcmol/l) hyperhomocysteinemia. More than half of the patients had HbA1c of greater than 7%, which indicated poor glycemic control (Table 2). 

**Table 2:** Categorical Parameters of Study Participants (n=188) HbA1c: hemoglobin A1c

Characteristics	N (%)
Gender	
Male	80 (42.66%)
Female	108 (57.44%)
Body mass index	
Healthy weight (18.5-24.9 kg/m^2^)	62 (32.97%)
Overweight (25.0-29.9 kg/m^2^)	88 (46.81%)
Obesity (30.0 kg/m^2^ and above)	38 (22.21%)
Serum homocysteine levels	
Normal (<15 mcmol/l)	75 (39.89%)
Moderate (15-30 mcmol/l)	24 (21.24%)
Intermediate (30-100 mcmol/l)	42 (37.17%)
Severe (>100 mcmol/l)	47 (41.59%)
Glycemic control	
Good glycemic control (HbA1c <7%)	31 (16.48%)
Poor glycemic control (HbA1c >7%)	157 (83.52%)
Hypertension	
Yes	79 (42.01%)
No	109 (57.98%)

The study revealed that the majority of the female patients, i.e. 37 (78.72%), had severe hyperhomocysteinemia (p<0.0001). Similarly, there was a direct correlation between HbA1c levels and serum Hcy. Severe hyperhomocysteinemia was found in over 80% of the patients with poor glycemic control, i.e. HbA1c >7% (p<0.0001). Furthermore, the durations of DMT2 and hypertension are both significantly associated with increased levels of homocysteinemia with p-values of <0.0001 and <0.0001, respectively. However, no association was found between hyperhomocysteinemia and BMI (Table 3). 

**Table 3:** The Association of Serum Homocysteine Levels With Gender, Body Mass Index, Glycemic Control, Duration of DMT2, and Hypertension (n=188) HbA1c: hemoglobin A1c; DMT2: diabetes mellitus type 2

Parameter(s)	Serum Homocysteine Levels				p-Value
	Normal (<15 mcmol/l)	Moderate (15-30 mcmol/l)	Intermediate (30-100 mcmol/l)	Severe (>100 mcmol/l)	
Gender					
Male (n=80)	52 (69.33%)	7 (29.17%)	11 (26.19%)	10 (21.28%)	<0.0001
Female (n=108)	23 (30.61%)	17 (70.83%)	31 (73.81%)	37 (78.72%)	
Body mass index					
Healthy weight (n=62)	24 (32%)	10 (41.7%)	11 (26.2%)	17 (36.2%)	0.524
Overweight (n=88)	38 (50.7%)	10 (41.7%)	23 (54.8%)	17 (36.2%)	
Obesity (n=38)	13 (17.3%)	4 (16.7%)	8 (19%)	13 (27.7%)	
Glycemic control					
HbA1c <7% (n=31)	8 (10.7%)	5 (20.8%)	10 (23.8%)	8 (17%)	<0.0001
HbA1c >7% (n=157)	67 (89.3%)	19 (79.2%)	32 (76.2%)	39 (83%)	
Duration of DMT2					
Less than five years (n=70)	42 (56%)	8 (33.3%)	13 (31%)	7 (14.9%)	<0.0001
Five years or greater (n=118)	33 (44%)	16 (66.7%)	29 (69%)	40 (85.1%)	
Hypertension					
Yes (n=79)	10 (13.3%)	10 (41.7%)	25 (59.5%)	34 (72.3%)	<0.0001
No (n=109)	65 (86.7%)	14 (58.3%)	17 (40.5%)	13 (27.7%)	

## Discussion

According to the results of our study, there was a direct association of serum Hcy with poor glycemic control as well as the duration of DMT2. Furthermore, the study also found that the majority of the patients with severe hyperhomocysteinemia were females. Finally, upon further assessment, it was revealed that hypertension was significantly correlated with increased levels of serum Hcy. Our study was comparable with the findings of Shaikh et al. [17]. In contrast to our study, Hoogeven et al. reported no significant link between serum Hcy and inadequate glycemic control [18]. Agha mohammadi et al. examined the association between HbA1c and serum Hcy in patients with DMT2 and failed to report any significant correlation between the two [19]. Pouwels et al. claimed the similar findings [20].

The present study failed to find an association between serum Hcy and the BMI. A study conducted in Kenya reported similar results and demonstrated no correlation between BMI and serum Hcy levels [21]. Other studies revealed similar results [22]. The link between obesity and hyperhomocysteinemia remains controversial. For future researches, the authors aim to evaluate the relationship between hypercholesteremia and hyperhomocysteinemia. 

We found a positive correlation between hypertension and increased levels of serum Hcy. Since hyperhomocysteinemia is known to damage the endothelial layer and also impact kidney function, this finding was not unexpected. Passaro et al. found similar associations [23]. However, some studies failed to find any association between hypertension and hyperhomocysteinemia [21]. 

It was found that the greater the duration of DMT2, the higher the levels of Hcy in the plasma. Coinciding results were observed by Sonkar et al. where the authors reported a significant association of plasma levels of Hcy and the duration and complications of DMT2 [24]. A study on patients presenting with diabetic retinopathy also reported a positive association of the duration of DMT2 with hyperhomocysteinemia [25]. Similarly, Mundu PA et al. found a direct relationship between duration of DMT2 and increased levels of serum Hcy [26].

In conclusion, we can suggest that hyperhomocysteinemia in patients with DMT2 directly depends on gender, BP, duration of DMT2, and poor glycemic control. However, the current study has some limitations. For instance, the current study was conducted only in a single institution and recruited only a limited number of patients owing to the resource and time restraints. Thus, the findings of the current study cannot be generalized to a larger population. Therefore, further studies with large and diversified sample sizes are recommended.

## Conclusions

The study revealed that increased levels of serum Hcy were associated with female gender, poor glycemic control (HbA1c >7%), BP, and duration of DMT2. However, the study failed to find an association between serum Hcy and the BMI. It is recommended that patients with poor glycemic control or those with the duration of DMT2 of more than five years must be regularly checked for hyperhomocysteinemia and renal function. 

Large-scale and multi-center studies are required in order to determine the validity of these findings. The current study suggests that patients with diabetes mellitus and hypertension are likely to have increased levels of Hcy and, therefore, must be regularly screened for hyperhomocysteinemia and subsequent renal failure. 
